# Ocular Effects of Glycyrrhizin at Acidic and Neutral pH

**DOI:** 10.3389/fcimb.2021.782063

**Published:** 2022-01-21

**Authors:** Mallika Somayajulu, Sharon A. McClellan, Denise A. Bessert, Ahalya Pitchaikannu, Linda D. Hazlett

**Affiliations:** Department of Ophthalmology, Visual and Anatomical Sciences, School of Medicine, Wayne State University, Detroit, MI, United States

**Keywords:** glycyrrhizin, keratitis, ocular surface, goblet cells, multi-drug resistance

## Abstract

**Purpose:**

To test the effects of acidic *vs*. neutral pH glycyrrhizin (GLY) on the unwounded and wounded normal mouse cornea and after infection with *Pseudomonas aeruginosa* isolates KEI 1025 and multidrug-resistant MDR9.

**Methods:**

Acidic or neutral GLY *vs*. phosphate-buffered saline (PBS) was topically applied to normal or wounded corneas of C57BL/6 mice. In unwounded corneas, goblet cells and corneal nerves were stained and quantitated. After wounding, corneas were fluorescein stained and photographed using a slit lamp. Mice also were infected with KEI 1025 or MDR9 and the protective effects of GLY pH evaluated comparatively.

**Results:**

In the unwounded cornea, application of acidic or neutral GLY *vs*. PBS reduced the number of bulbar conjunctival goblet cells but did not alter corneal nerve density. Similar application of GLY to scarified corneas delayed wound closure. After KEI 1025 infection, none of the GLY *vs*. PBS-treated corneas perforated; GLY treatment also decreased plate count (neutral pH more effective) and reduced MPO and several cytokines. Similarly, for MDR9, GLY at either pH was protective and also enhanced the effects of moxifloxacin to which MDR9 is resistant.

**Conclusion:**

Acidic or neutral pH GLY decreased goblet cell number but had no effect on nerve density. After corneal wounding, GLY at either pH (1) delayed wound closure and, (2) after infection, decreased keratitis when used alone or in combination with moxifloxacin. Neutral pH did not alter the therapeutic effect of GLY and would be preferred if used clinically.

## Introduction

Glycyrrhizin (GLY), a derivative of the licorice root, is used in traditional medicine in many parts of the world due to its nutritional and therapeutic properties (Isbrucker and Burdock, 2006). Structurally, it is a triterpenoid saponin with anti-inflammatory properties (Yang et al., 2017). In this regard, GLY directly binds to high-mobility group box 1 (HMGB1) and inhibits its chemoattractant and mitogenic activity (Mollica et al., 2007). GLY is approved by the US Food and Drug Administration (FDA) and used clinically to treat patients with viral hepatitis C (Arase et al., 1997), severe acute respiratory syndrome (SARS) virus (Sinha et al., 2020), and HIV (Hattori et al., 1989). Studies from our laboratory have shown the effectiveness of GLY against *Pseudomonas aeruginosa* keratitis using a cytotoxic strain 19660 (ATCC) (Ekanayaka et al., 2016), a non-ocular multidrug-resistant clinical isolate MDR9 (Hazlett et al., 2019), and an ocular non-cytotoxic clinical isolate, KEI 1025 (Ekanayaka et al., 2016; Ekanayaka et al., 2018). In addition, we showed that GLY enhanced the efficacy of the antibiotic, tobramycin, in a KEI 1025-induced model of bacterial keratitis (Ekanayaka et al., 2018). GLY also enhanced the effects of ciprofloxacin to which MDR9 is resistant, by reducing the minimum inhibitory concentration twofold (Hazlett et al., 2019). Others also showed that GLY enhances bioavailability of antibiotics against *P. aeruginosa in vitro* (Chakotiya et al., 2016). Despite the fact that all of these studies have shown effectiveness of GLY alone and in combination with antibiotics to treat keratitis, we have not examined if GLY induces adverse events in the cornea at acidic pH, which we used for those studies. Neither was pH considered in other animal models, in which GLY is protective, including in lung (Ni et al., 2011) and cerebral ischemic (Gong et al., 2014) injuries, colitis (Liu et al., 2011), and sepsis (Wang et al., 2013). In contrast, in other studies, ophthalmic solutions containing GLY as nanocarriers at neutral pH were used to treat *Staphylococcus aureus* infection in rabbits (Song et al., 2020; Zhang et al., 2021) and corneal wounds in diabetic mice (Hou et al., 2021a). In this regard, pilot clinical studies have investigated the efficacy of GLY in ophthalmic solutions (all at neutral pH) to treat blepharitis (Mencucci et al., 2013) and dry eye disease (Burillon et al., 2018).

Thus, the objectives of this study are to test if the pH of GLY (acidic or neutral) has effects on the normal unwounded, uninfected cornea and conjunctiva, and after corneal wounding. We also tested if the pH of GLY affected its ability to protect the cornea and whether GLY at neutral pH was able to potentiate an antibiotic similarly to that observed with acidic GLY.

Evidence is provided that topical application of GLY at either pH *vs*. phosphate-buffered saline (PBS) reduced the number of goblet cells in the bulbar conjunctiva but did not affect nerve density in the normal, unwounded cornea. In scarified corneas, at either pH, GLY led to delayed wound closure at 6 h compared to PBS. After infection with KEI 1025, neutral *vs*. acidic pH GLY was significantly better at reducing bacterial plate count; for MDR9, neutral and acidic pH GLY, when combined with moxifloxacin, were similar in effect at reducing plate count and the neutrophil infiltrate.

## Materials and Methods

### Mice

Female C57BL/6 mice, 8 weeks of age, were purchased from the Jackson Laboratory (Bar Harbor, ME) and housed in accordance with the National Institutes of Health guidelines. Mice were humanely treated and in compliance with both the ARVO Statement for the Use of Animals in Ophthalmic and Vision Research and the Institutional Animal Care and Use Committee of Wayne State University (IACUC-21-04-3499).

### GLY Topical Application

Uninfected, unwounded, normal corneas (both eyes) received topical application of either acidic (pH 3.5–3.7) or neutral (pH 7.2–7.4) GLY (100 µg in 5 µl PBS, Sigma Aldrich, St. Louis, MO) or PBS (control) each day (5 µl drop, 2×/day) for up to 4 days and tested at 3 and/or 5 days. In another experiment, a 5-µl drop of acidic or neutral GLY or PBS was topically applied to the left cornea of C57BL/6 mice 30 min after scarification of normal cornea and tested after 6 and 24 h. For infection studies, infected corneas were topically treated with PBS or acidic or neutral GLY (5 µl drop) once 6 h post-infection (p.i.) and twice daily for up to 4 days and tested at 3 and/or 5 days. The GLY concentration was chosen based on previously published reports (Ekanayaka et al., 2018).

### Goblet Cell Staining

To stain conjunctival (bulbar) goblet cells, whole eyes (uninfected and unwounded, n = 3/group) received acidic or neutral GLY *vs*. PBS topically. Both eyes were enucleated 5 days later, fixed in alcoholic Z fix (provided by Excalibur Pathologies Inc., Norman, OK, USA), and sent to Excalibur Pathologies for paraffin embedding and sectioning. Periodic acid–Schiff (PAS) staining (stains vicinal glycols) was used to visualize the cells. The number of goblet cells per section were counted from three mice in each group (25 sections total/each treatment), and representative images were photographed using a Leica DM4000 microscope.

### Corneal Nerve Staining

Nerve staining was done as described before (He and Bazan, 2016) on normal, uninfected, and unwounded corneas (n = 3/group) at day 5 after mice received topical acidic or neutral GLY or PBS for 4 days. Briefly, corneas were excised, fixed in 2% paraformaldehyde for 1 h, and washed with 0.1 M phosphate buffer (PB) containing 0.1% bovine serum albumin (BSA) (PB-BSA). To block non-specific binding, corneas were individually placed in a 96-well plate and incubated with blocking solution containing 10% normal donkey serum plus 0.3% Triton X-100 in PB-BSA for 1 h at room temperature. Next, the corneal tissue was incubated with mouse monoclonal anti-βIII-tubulin antibody (BioLegend, San Diego, CA, USA) in blocking solution (1:300) for 72 h at 4°C. After three washes with PB-BSA, they were incubated with donkey antimouse IgG Alexa Fluor 594 (Jackson ImmunoResearch, West Grove, PA, USA) antibody for 24 h at 4°C and then washed with PB-BSA. Four radial cuts were made to the cornea before mounting on a slide using VECTASHIELD (Vector Laboratories Inc., Burlingame, CA, USA). Slides were imaged using a Leica TCS SP8 microscope. Images were processed, and green pseudo-colored images were generated using Adobe Photoshop CS6 version 13.0. To quantitate nerve density, images in tiff format (taken on the Leica SP8 confocal microscope) were converted into binary images using Image J version 1.49b software. Each binary image was then inverted, divided into nine sections, and histograms were recorded for each section using Adobe Photoshop CS6 version 13.0. Nerve density was measured from three mice in each group (8 images × 9 sections = 72 sections/each treatment).

### Corneal Wound Healing and Fluorescein Staining

To determine the effects of acidic or neutral GLY *vs*. PBS on corneal wound healing, anesthetized mice (n = 3/group/time) were placed beneath a stereoscopic microscope at 40× magnification, and the left cornea was wounded by making three 1-mm incisions using a sterile 25 5/8 gauge needle, penetrating the epithelial cell basal lamina and into the superficial corneal stroma. Acidic or neutral GLY or PBS was topically applied to the left cornea 30 min after scarification. After 6 and 24 h, a 5 μl drop of fluorescein (sterile 0.25% fluorescein sodium, Accutome, Malvern PA) was topically applied to the eye surface, allowed to penetrate for 3 min, blotted, and eyes photographed using a slit lamp with a cobalt blue filter.

### Bacterial Culture and Infection

*Pseudomonas aeruginosa* strains, KEI 1025, an ocular non-cytotoxic clinical isolate (Kresge Eye Institute, Detroit, MI), and MDR9, a non-ocular multidrug-resistant clinical isolate (from sputum; Detroit Medical Center, Detroit, MI, USA), were grown in peptone tryptic soy broth (PTSB) medium in a rotary shaker water bath at 37°C and 150 rpm for 18 h to an optical density (measured at 540 nm) between 1.3 and 1.8. Bacterial cultures were centrifuged at 5,500 *g* for 10 min. Pellets were washed once with sterile saline, recentrifuged, resuspended, and diluted in sterile saline. Mice were infected as described before (Moon et al., 1988; Ekanayaka et al., 2018). Briefly, mice were anesthetized with ether and placed under a stereoscopic microscope at 40× magnification. The left cornea was scarified, and 5 μl containing 1 × 10^7^ colony-forming units (CFU)/μl (KEI 1025) or 1 × 10^8^ CFU/μl (MDR9) of the bacterial suspension was applied topically.

### Ocular Response to Bacterial Infection

Clinical scores (n = 5/group/time/experiment) were designated as follows: 0 = clear or slight opacity, partially or fully covering the pupil; +1 = slight opacity, fully covering the anterior segment; +2 = dense opacity, partially or fully covering the pupil; +3 = dense opacity, covering the entire anterior segment; and +4 = corneal perforation or phthisis (Moon et al., 1988). Each mouse was scored in masked fashion at 1, 3, and 5 days p.i. for statistical comparison and photographed (5 days p.i.) with a slit lamp to illustrate disease.

### GLY Treatment After KEI 1025 or MDR9 Infection

To study the effects of the pH of GLY on *P. aeruginosa* keratitis, using KEI 1025, acidic or neutral GLY (100 μg in 5 µl PBS) or PBS was topically applied 6 h p.i. and twice daily from 1 to 4 days p.i. (Ekanayaka et al., 2018). To study if pH was important for bacterial killing, the pH of PBS was adjusted to either acidic or neutral as a control for acidic *vs*. neutral GLY effects. For this experiment, mice received topical applications of PBS adjusted to acidic (3.75) or neutral (7.2) pH, at 6 h after KEI 1025 infection (as described above). Another experiment was done using MDR9 to determine if the pH of GLY affected its ability to augment the efficacy of the antibiotic, moxifloxacin. For the latter experiment, treatment was delayed until 18 h after infection (Hazlett et al., 2019). Mice (*n* = 5/group/time/treatment) were infected with MDR9 as described above. Beginning at 18 h p.i., corneas were treated topically with acidic or neutral GLY (100 μg/dose) plus moxifloxacin (12.5 μg/dose) to which MDR9 is resistant (data not shown) or PBS or moxifloxacin alone twice daily through 4 days p.i.

### Myeloperoxidase Assay

An myeloperoxidase (MPO) assay was used to quantitate neutrophils in corneas infected with KEI 1025 or MDR9 (*n* = 5/group/time/experiment). Individual corneas were excised at 3 and 5 days p.i. and homogenized in 1 ml of 50 mM phosphate buffer (pH 6.0) containing 0.5% hexadecyltrimethylammonium bromide (HTAB). Samples were freeze–thawed four times and centrifuged. A 100-μl aliquot of the supernatant was added to 2.9 ml of 50 mM phosphate buffer containing *o*-dianisidine dihydrochloride (16.7 mg/ml, Sigma-Aldrich) and hydrogen peroxide (0.0005%). The change in absorbency was monitored at 460 nm for 4 min at 30-s intervals. The slope of the line was determined for each sample and used to calculate units of MPO/cornea. One unit of MPO activity is equivalent to ∼2 × 10^5^ neutrophils (Williams et al., 1982; Ekanayaka et al., 2016).

### Quantification of Viable Bacteria

Viable bacteria were quantitated in the cornea of KEI 1025- and MDR9-infected mice at 3 and 5 days p.i. (*n* = 5/group/time/experiment). Each cornea was homogenized in 1 ml of sterile saline (0.85% NaCl, pH 7.4) containing 0.25% BSA. A 100-μl aliquot of the corneal homogenate was serially diluted (1:10) in sterile saline containing 0.25% BSA, and selected dilutions were plated in triplicate on selective culture medium (Difco Pseudomonas Isolation Agar, BD Biosciences, Inc., Franklin Lakes, NJ, USA). Plates were incubated overnight at 37°C and viable bacteria manually counted. Results are reported as log_10_ CFU/cornea ± SEM (Hobden et al., 1997; Ekanayaka et al., 2016).

### RT-PCR

Total RNA was isolated (RNA STAT-60; Tel-Test, Friendswood, TX) from KEI 1025-infected corneas treated with acidic or neutral GLY *vs*. PBS (n = 5/group) at 5 days p.i. as reported before (Huang et al., 2005). One microgram of each RNA sample was reverse transcribed using Moloney-murine leukemia virus (M-MLV) reverse transcriptase (Invitrogen, Carlsbad, CA) to produce a cDNA template. cDNA products were diluted 1:20 with diethylpyrocarbonate (DEPC)-treated water, and a 2-μl aliquot of diluted cDNA was used for the reverse transcription PCR (RT-PCR). SYBR green/fluorescein PCR master mix (Bio-Rad Laboratories, Richmond, CA) and primer concentrations of 10 μM were used in a total 10 μl volume. After a preprogrammed hot start cycle (3 min at 95°C), the parameters used for PCR amplification were 15 s at 95°C and 60 s at 60°C with the cycles repeated 45 times. Levels of high mobility group box 1 (HMGB1), interleukin (IL), 1β, C-X-C motif chemokine ligand 2 (CXCL2), and tumor necrosis factor (TNF) α were tested by real-time RT-PCR (CFX Connect real-time PCR detection system; Bio-Rad Laboratories). The fold differences in gene expression were calculated relative to control and normalized to housekeeping gene 18S rRNA (mouse) and expressed as the relative mRNA concentration ± SEM. Primer pair sequences used are shown in **Table 1**.

**Table 1:** Nucleotide sequence of the specific primers used for PCR amplification (mouse).

Gene	Nucleotide Sequence	Primer	GenBank
*18s*	5′-GTA ACC CGT TGA ACC CCA TT-3′	F	NR_003278.3
	5′- CCA TCC AAT CGG TAG CG-3′	R	
*Cxcl2*	5′- TGT CAA TGC CTG AAG ACC CTG CC -3′	F	NM_010439.3
	5′- AAC TTT TTG ACC GCC CTT GAG AGT GG -3′	R	
*Hmgb1*	5′- TGG CAA AGG CTG ACA AGG CTC -3′	F	NM_008361.3
	5′- GGA TGC TCG CCT TTG ATT TTG G -3′	R	
*Il-1β*	5′- TGT CCT CAT CCT GGA AGG TCC ACG -3′	F	NM_009140.2
	5′- TGT CCT CAT CCT GGA AGG TCC ACG -3′	R	
*Tnf-α*	5′- ACC CTC ACA CTC AGA TCA TCT T-3′	F	NM_013693.2
	5′- GGT TGT CTT TGA GAT CCA TGC-3′	R	

F, forward; R, reverse.

### ELISA

ELISA kits were used to measure protein levels of HMGB1 (Chondrex, Inc., Redmond, WA), CXCL2, and TNF-α (R&D Systems Inc., Minneapolis, MN). KEI 1025-infected corneas from acidic or neutral pH GLY *vs*. PBS-treated C57BL/6 mice were harvested at 3 and 5 days p.i. (n = 5/group/time). To quantify CXCL2, individual corneas were homogenized in 1 ml of 50 mM potassium phosphate buffer (pH 6.0) containing 0.5% hexadecyltrimethylammonium bromide (HTAB, Millipore Sigma, Madison, WI). To measure HMGB1 and TNF-α, corneas were homogenized in 500 µl of PBS containing 0.1% Tween 20 and protease inhibitors. Corneal homogenates were centrifuged at 12,000 *g* for 10 min. A 50-μl aliquot of each supernatant, diluted 1:5 for HMGB1, 1:2 for CXCL2, and undiluted for TNF-α, was assayed in duplicate. All assays were run per the manufacturers’ protocol.

### Statistical Analysis

A one-way ANOVA followed by the Bonferroni’s multiple comparison test (GraphPad Prism) was used for analysis when comparing three or more groups (goblet cells, nerve density, RT-PCR, MPO, plate count, clinical score, and ELISA). For each test, *p* < 0.05 was considered significant, and data are shown as mean ± SEM. All experiments were repeated at least once to ensure reproducibility.

## Results

### Effects of GLY pH on Goblet Cells

**Figure 1:**
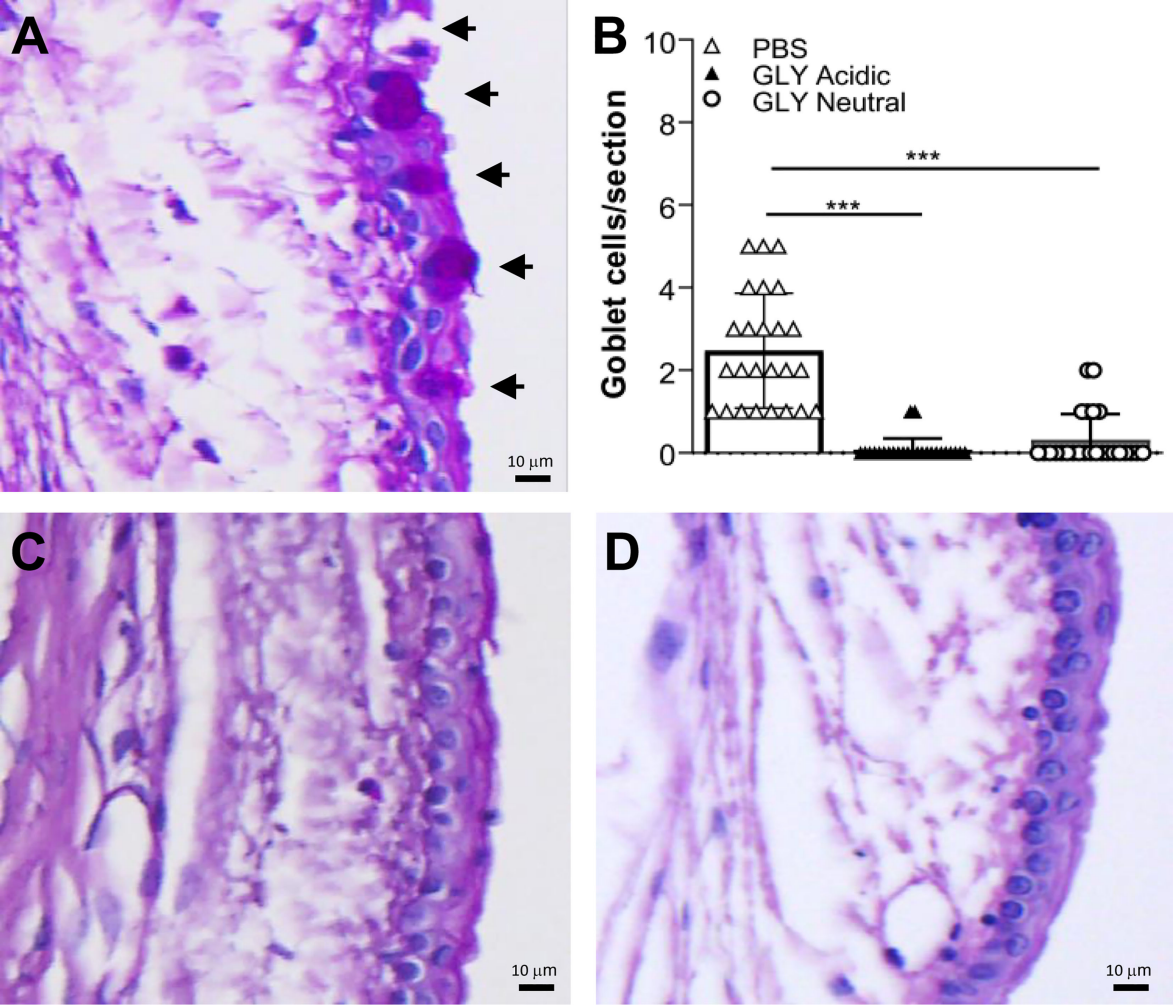
Bulbar goblet cells (arrows) in normal conjunctiva (no corneal wound) in eyes that received topical application of PBS **(A)**, or acidic **(C)**, or neutral GLY **(D)** for 4 days and tested on day 5. Acidic **(C)** or neutral GLY **(D)** *vs*. PBS **(A)** showed significantly reduced goblet cell number **(B)** on day 5. Data are expressed as mean ± SEM (***p < 0.001, n = 3/group). Scale bar = 10 μm.

PAS positively stained bulbar conjunctival goblet cells were examined and quantitated (**Figures 1A–D**) in the conjunctiva of normal, unwounded eyes (cornea) at 5 days after acidic (**Figure 1C**) or neutral (**Figure 1D**) GLY *vs*. PBS (**Figure 1A**) application. PAS positively stained goblet cells were routinely observed after PBS (**Figure 1A**, arrows) application but were rarely seen after either acidic (**Figure 1C**) or neutral (**Figure 1D**) GLY application. Both acidic and neutral GLY application significantly reduced the number of PAS-positive cells *vs*. PBS (**Figure 1B**, p < 0.001 for both).

### Effect of GLY pH on Corneal Nerves

**Figure 2:**
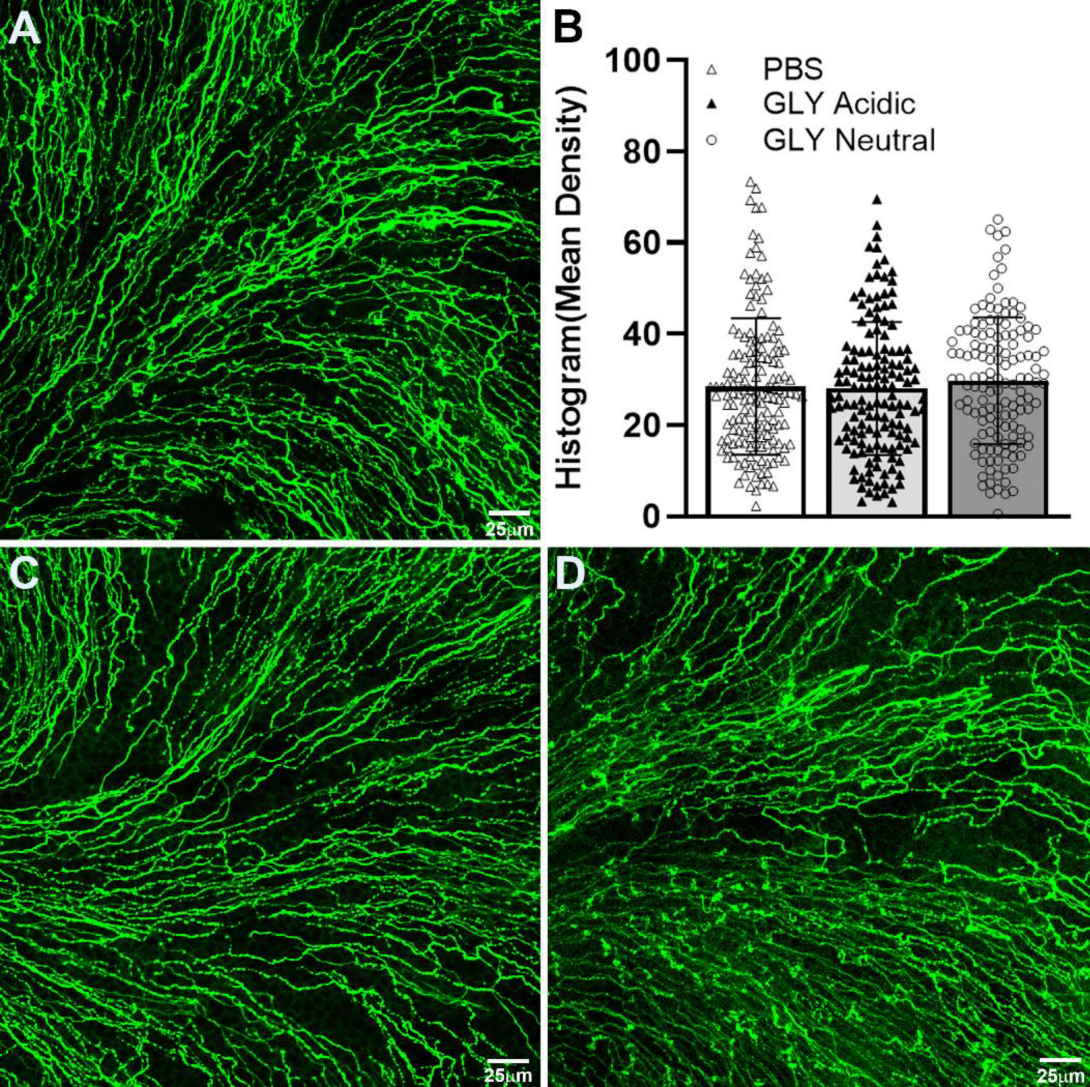
Confocal images of corneal whole mounts stained with β-tubulin to visualize corneal nerves. Mice received topical PBS **(A)**, acidic **(C)**, or neutral **(D)** GLY onto the normal, unwounded cornea for 4 days and were tested on day 5. **(B)** Histogram analysis showed no differences in nerve fiber density among the three groups. Data are expressed as mean ± SEM (n = 3/group). Scale bar = 25 μm.

Corneal nerves were stained at 5 days after GLY (acidic and neutral) *vs*. PBS application (**Figures 2A–D**) and nerve density was examined and quantitated in the normal cornea. No alterations in the assembly of whorl patterns of subbasal nerve fiber were observed among the three groups (data not shown) at day 5. No difference in nerve density between GLY at acidic (**Figure 2C**) or neutral (**Figure 2D**) pH *vs*. PBS (**Figure 2A**) was detected using histogram analysis (**Figure 2B**).

### Effects of GLY pH on Wound Healing

**Figure 3:**
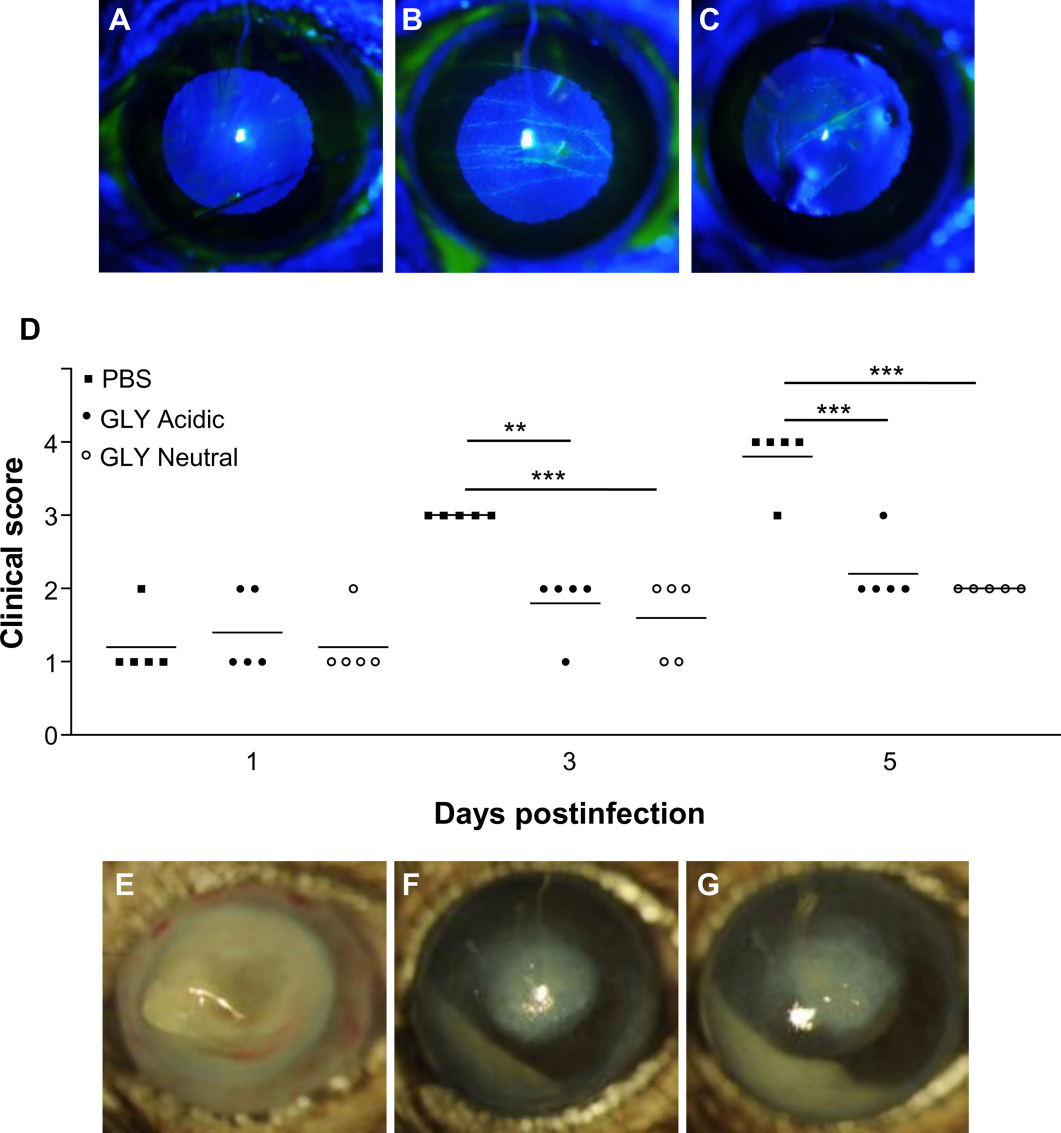
Slit lamp photographs of fluorescein-stained scarified corneas (n = 3/group/time) that received PBS **(A)**, acidic **(B)**, or neutral **(C)** GLY. Wound closure was delayed at 6 h by GLY **(B, C)** *vs*. PBS **(A)** application where wounds were undetectable. **(D–G)** Effects of GLY pH on corneas infected with KEI 1025. **(D)** Clinical scores after treatment (beginning at 6 h p.i.) with PBS or acidic or neutral GLY at 1, 3, and 5 days p.i. Lower clinical scores were observed after treatment with acidic or neutral GLY *vs*. PBS at both 3 and 5 days p.i. **(E–G)** Slit lamp photographs from PBS **(E)**, or acidic **(F)** or neutral **(G)** GLY-treated corneas at 5 days p.i. show perforation only in the PBS-treated group **(E)** (**p < 0.01, ***p < 0.001, n = 5/group/time).

**Figures 3A–C** show fluorescein-stained normal corneas at 6 h after wounding. GLY application at both acidic (**Figure 3B**) and neutral (**Figure 3C**) pH *vs*. PBS (**Figure 3A**) showed fluorescein staining, at 6 h after wounding, indicating delayed wound closure. All corneas that received PBS after wounding had closed at this time. By 24 h after wounding, no differences in wound closure among the three groups was detected, and all corneas appeared similar (data not shown).

### Effect of GLY pH on Infection With KEI 1025

**Figure 4:**
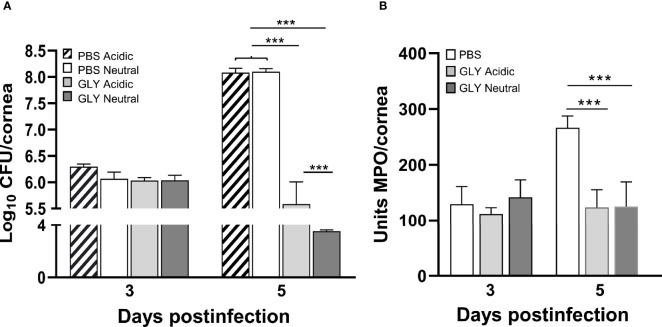
Bacterial plate count and MPO in corneas infected with KEI 1025. **(A)** Bacterial plate counts at 3 and 5 days p.i. after acidic or neutral GLY *vs*. PBS (adjusted to acidic or neutral pH) treatment show significant reduction only at 5 days p.i., and GLY at neutral pH was slightly but significantly better than acidic pH **(B)**. MPO levels measured at 3 and 5 days p.i. after acidic or neutral GLY *vs*. PBS show significant reduction only at 5 days p.i. Data are expressed as mean ± SEM (***p < 0.001, n = 5/group/time).

**Figure 3D** shows clinical scores of KEI 1025-infected mice after treatment with acidic or neutral pH GLY *vs*. PBS. As in the past work (Ekanayaka et al., 2018), treatment began at 6 h after infection. Significantly less corneal disease with lower clinical scores was seen after application of either acidic (p < 0.01, p < 0.001) or neutral GLY (p < 0.001, p < 0.001) *vs*. PBS at 3 and 5 days p.i., respectively. Photographs taken with a slit lamp of representative eyes from PBS (**Figure 3E**), acidic (**Figure 3F**), and neutral (**Figure 3G**) GLY-treated mice at 5 days p.i. show that the majority of eyes treated with PBS had perforated and opacity was pronounced. No perforation was observed in the GLY-treated eyes that also exhibited reduced opacity confined to the central cornea with hypopyon visible in the anterior chamber. To rule out the possibility that the pH of GLY was responsible for bacterial killing, PBS was adjusted to neutral or acidic pH. Viable bacterial plate count (**Figure 4A**) showed no significant differences between acidic or neutral GLY *vs*. either PBS group at 3 days p.i. By 5 days p.i., viable bacterial counts were significantly reduced in both acidic (p < 0.001) and neutral (p < 0.001) GLY *vs*. either PBS groups. At this time, although both neutral and acidic GLY reduced plate count significantly compared to controls, neutral (p < 0.001) *vs*. acidic GLY treatment was significantly less (about 1.5 logs). There was no difference in plate count between acidic or neutral pH PBS. MPO activity (**Figure 4B**) did not differ between acidic or neutral GLY *vs*. the PBS-treated group at 3 days p.i. However, by 5 days p.i., both acidic (p < 0.001) and neutral (p < 0.001) GLY *vs*. PBS reduced MPO similarly when compared to PBS.

### Effects of GLY pH on Cytokines

**Figure 5:**
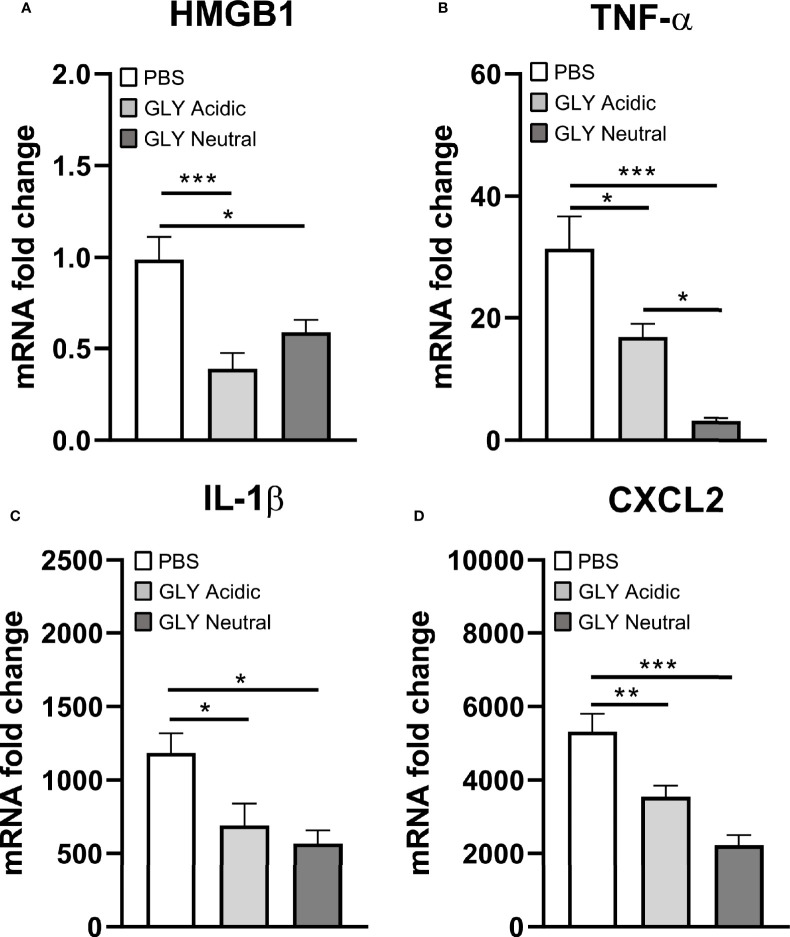
Effects of GLY pH on mRNA levels of cytokines in corneas infected with KEI 1025 and treated with PBS or acidic or neutral GLY. Significantly reduced mRNA expression for HMGB1 **(A)**, TNF-α **(B)**, IL-1β **(C)**, and CXCL2 **(D)** after acidic or neutral GLY *vs*. PBS treatment was observed at 5 days p.i. Data are expressed as mean ± SEM (*p < 0.05, **p < 0.01, ***p < 0.001, n = 5/group/time).

**Figure 6:**
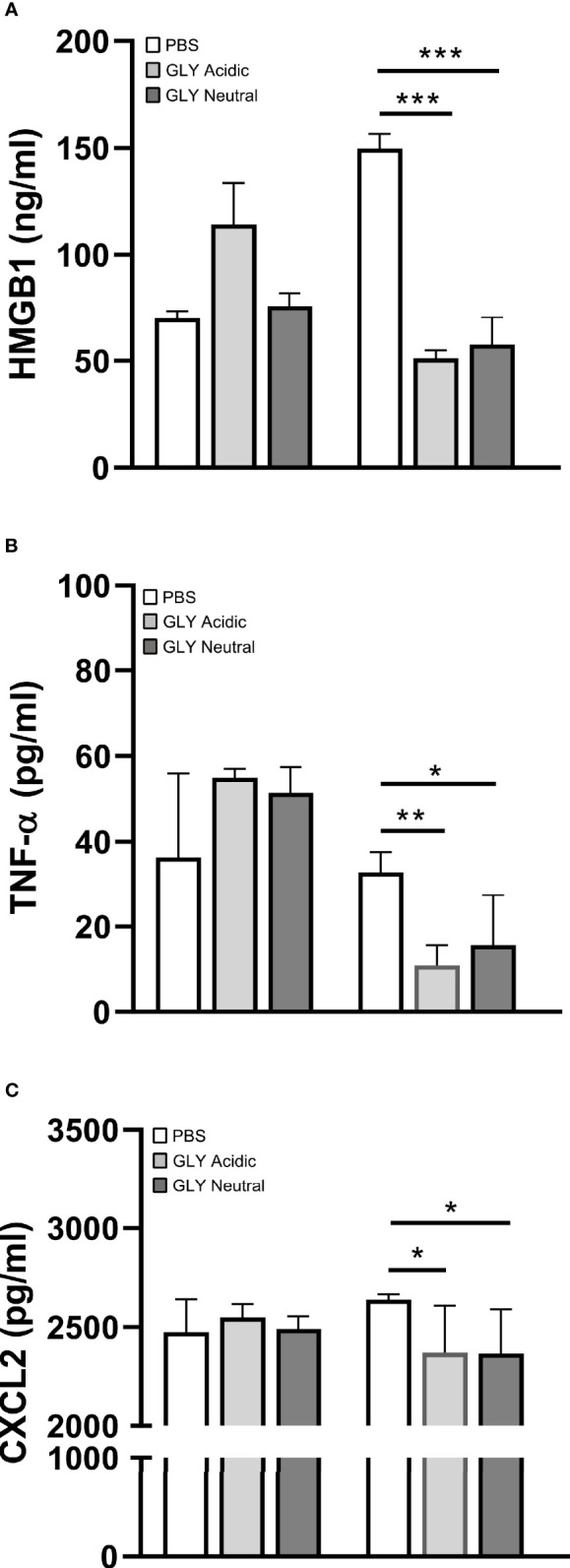
Effects of GLY pH on protein levels of cytokines in corneas infected with KEI 1025 and treated with PBS or acidic or neutral GLY (began 6 h after infection). ELISA detected protein levels of HMGB1 **(A)**, TNF-α **(B)**, and CXCL2 **(C)** at 3 and 5 days p.i. Significantly reduced protein levels for all three cytokines after acidic or neutral GLY *vs*. PBS treatment were observed only at 5 days p.i. Data are expressed as mean ± SEM (*p < 0.05, **p < 0.01, ***p < 0.001, n = 5/group/time).

**Figure 5** shows the relative mRNA levels for HMGB1 (**Figure 5A**), TNF-α (**Figure 5B**), IL-1β (**Figure 5C**), and CXCL2 (**Figure 5D**) at 5 days after KEI 1025 infection and treatment with acidic or neutral GLY or PBS. Acidic and neutral GLY *vs*. PBS treatment significantly reduced mRNA levels for HMGB1 (acidic p < 0.001, neutral p < 0.05), TNF-α (acidic p < 0.05, neutral p < 0.001), IL-1β (p < 0.05 for both) and CXCL2 (acidic p < 0.01, neutral p < 0.001). Only for TNF-α mRNA levels, neutral *vs*. acidic GLY treatment was significantly more effective (p < 0.05, **Figure 5B**). Protein analysis (**Figure 6**) showed no significant differences in HMGB1 (**Figure 6A**), TNF-α (**Figure 6B**), and CXCL2 (**Figure 6C**) levels between PBS or acidic or neutral GLY-treated groups at 3 days p.i. However, at 5 days p.i., protein levels were significantly reduced in both acidic and neutral GLY *vs*. PBS groups for HMGB1 (p < 0.001 for both), TNF-α (acidic, p < 0.01, neutral p < 0.05), and CXCL2 (p < 0.05 for both).

### Effect of GLY pH on MDR9

**Figure 7:**
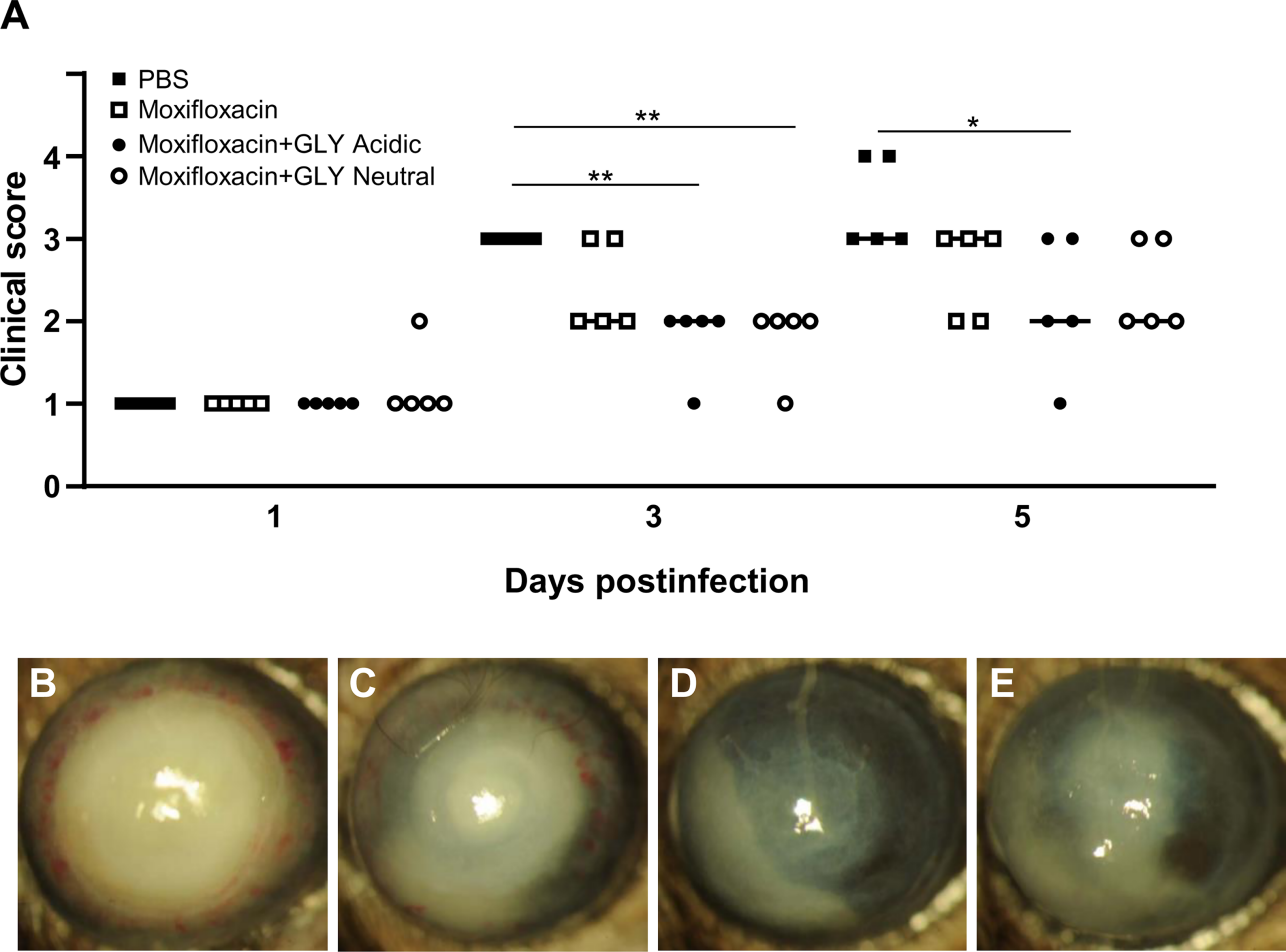
Effects of GLY pH on corneas infected with MDR9. **(A)** Clinical scores at 1, 3, and 5 days p.i. after treatment with PBS, moxifloxacin, and acidic or neutral GLY plus moxifloxacin (began 18 h after infection). Similar lower clinical scores were observed at 3 and 5 days p.i. when moxifloxacin was combined with either acidic or neutral GLY *vs* PBS. Slit lamp photographs from PBS **(B)**, moxifloxacin **(C)**, moxifloxacin plus acidic **(D)**, or neutral **(E)** GLY-treated corneas at 5 days p.i. show significantly reduced opacity confined over the pupil, with hypopyon in the anterior chamber in mice treated with moxifloxacin together with either acidic or neutral GLY *vs*. PBS (*p < 0.05, **p < 0.01, n = 5/group/time).

**Figure 8:**
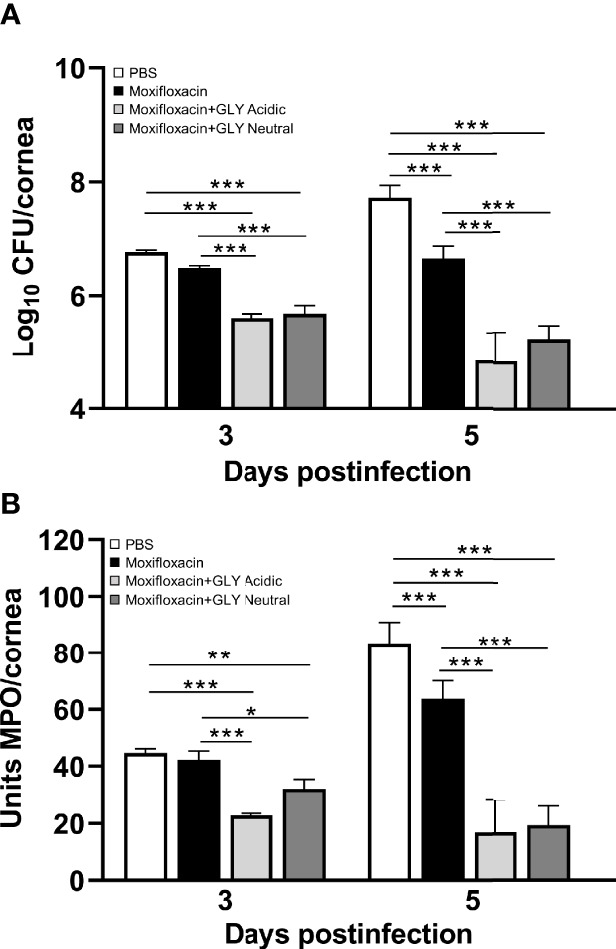
Effects of GLY pH on corneas infected with MDR9. **(A)** Bacterial plate counts and **(B)** MPO levels at 3 and 5 days p.i. after treatment with moxifloxacin plus acidic or neutral GLY *vs*. PBS or moxifloxacin alone were significantly reduced. Data are expressed as mean ± SEM (*p < 0.05, **p < 0.01, ***p < 0.001, n = 5/group/time).

Clinical scores of mice infected with MDR9 and 18 h later treated with moxifloxacin ± acidic or neutral GLY or PBS are shown in **Figure 7A**. At 3 days p.i., lower clinical scores in mice treated with moxifloxacin (MDR 9 is resistant, data not shown) plus acidic (p < 0.01) or neutral (p < 0.01) GLY *vs*. PBS but not between PBS and moxifloxacin alone were observed. However, at 5 days p.i., acidic GLY plus moxifloxacin-treated corneas showed significantly lower clinical scores (p < 0.05) *vs*. PBS. Photographs of eyes taken with a slit lamp at 5 days p.i. from PBS (**Figure 7B**) showed greater opacity and corneal thinning compared to moxifloxacin-treated (**Figure 7C**) eyes. Combining acidic (**Figure 7D**) or neutral (**Figure 7E**) GLY with moxifloxacin resulted in decreased opacity (central cornea, over pupil, and visible hypopyon) compared to PBS (**Figure 7B**) or moxifloxacin (**Figure 7C**) alone. Viable bacterial plate counts (**Figure 8A**) showed that treatment with acidic or neutral GLY plus moxifloxacin significantly decreased bacterial load at 3 days p.i., compared to either PBS (p < 0.001 for both) or moxifloxacin (p < 0.001 for both). At 5 days p.i., bacterial counts were further significantly reduced in both acidic and neutral GLY plus moxifloxacin-treated groups compared to PBS (p < 0.001for both) or moxifloxacin (p < 0.001). Some reduction in bacterial counts also was observed when comparing PBS to moxifloxacin alone (p < 0.001) at 5 days p.i. (**Figure 8A**). **Figure 8B** shows MPO activity was significantly reduced in both acidic and neutral GLY plus moxifloxacin-treated groups compared with PBS (acidic p < 0.001, neutral p < 0.01) or moxifloxacin alone (acidic p < 0.001; neutral p < 0.05) at 3 days p.i. By 5 days p.i, MPO was significantly reduced further for acidic or neutral GLY plus moxifloxacin groups compared to either PBS or moxifloxacin alone. Some reduction in MPO also was observed when comparing PBS to moxifloxacin alone (p < 0.001) at 5 days p.i. (**Figure 8B**).

## Discussion

GLY is FDA approved (Omar et al., 2012) and has both anti-inflammatory and antimicrobial properties (Asl and Hosseinzadeh, 2008). For example, it has shown efficacy when used topically in the clinic to treat patients with blepharitis (Mencucci et al., 2013) and dry eye (Burillon et al., 2018) disease. These two clinical pilot studies, one using a 5% GLY ophthalmic solution to treat blepharitis and the other using eye drops containing 2.5% GLY to treat dry eye patients, showed that GLY at a neutralized pH was well tolerated in both healthy volunteers (normal) and in patients. Because patients were being treated, only non-invasive approaches could be used to assess any adverse consequences of GLY use. However, animal models provide the ability when scientifically and humanely justified, to use invasive and non-invasive testing. Thus, to determine whether the pH of GLY correlated with adverse ocular changes, we tested its effect at acidic and neutral pH on the normal, uninfected ocular surface. Examining the conjunctiva, we observed a significant reduction in bulbar conjunctival goblet cells after 4 days of either acidic or neutral GLY *vs*. PBS application. Unfortunately, there is very little data on the effects of GLY on goblet cells in the normal eye at either pH. However, it was reported in a mouse model of goblet cell hyperplasia in the airway epithelium that GLY (pH not indicated) reduced goblet cells (Nishimoto et al., 2010). GLY also decreased mucus production by attenuating mRNA and protein levels of *MUC5AC* and inhibiting *MUC5AC* gene transcription (Nishimoto et al., 2010). Similarly, another study using a mouse model of asthma showed that GLY (pH not specified) administered *via* orogastric gavage reduced goblet cells in the trachea (Hocaoglu et al., 2011). These are all consistent with the data reported herein.

Our data on corneal nerve density used the normal, unwounded cornea treated with GLY at either pH and showed no differences when compared with PBS treatment. These data differ from numerous studies showing that GLY is neuroprotective; however, all of those studies used models of diseases. For example, examining wound healing in the diabetic mouse cornea, a dipotassium salt of GLY (pH not specified) significantly increased subbasal nerve density (Hou et al., 2021b). Additionally, in the diabetic mouse cornea, a combination of GLY as a nanocarrier encapsulating genistein (pH 7.2) showed increased subbasal nerve density and accelerated corneal nerve regeneration (Hou et al., 2021a). GLY also showed neuroprotective capability in other disease models, including traumatic brain injury (Okuma et al., 2014), epilepsy (Li et al., 2019), Alzheimer’s disease (Abdel Bar et al., 2019), Parkinson’s disease (Ojha et al., 2016), and multiple sclerosis (Sun et al., 2018).

When GLY at both acidic and neutral pH, was applied to the scarified normal, uninfected cornea, delayed wound closure was observed at 6 h, but not at 24 h, when compared to PBS. In contrast, in diabetic corneas that received a 2-mm trephine wound, GLY promoted regeneration of the corneal epithelium (Hou et al., 2021b), and closure was observed at 24 h *vs*. control (Zhou et al., 2020; Hou et al., 2021b). In the skin, licorice containing creams are commonly used to treat wounds (Mostafa et al., 2004, Ciganović et al., 2019). In this regard, studies in rabbits (Zaki et al., 2005) and guinea pigs (Hanafi et al., 2018) receiving surgical blade induced full-thickness wounds showed that 10% licorice cream provided more rapid healing than control treatment (12–14 days). Our data are disparate from these studies, but we hypothesize that this may be due to various factors such as species and/or tissue differences, the type of wounding (depth and breadth), dose of GLY, duration of application, and the time points at which wound healing was examined. For example, in our study, the wound consisted of three 1-mm scratches to the central cornea using a 25^5/8^ gauge needle, which penetrated just below the epithelial basal lamina into the superficial stroma, unlike the large sized wound of the 2-mm trephined cornea. In addition, we tested corneal wound healing earlier after GLY application. Other studies used much larger areas of wounding, including a 2-mm trephine wound (cornea) or full thickness skin wounds (rabbit and guinea pig) and looked at wound healing at 24 h or days later.

In a disease (infection) model in cornea, GLY (acidic pH) showed efficacy when used topically in an experimental mouse model of *P. aeruginosa* keratitis (Ekanayaka et al., 2018). The current study sought to determine whether the acidic pH was critical for protection. Using a non-drug-resistant clinical isolate KEI 1025, we found that GLY-treated corneas responded similarly in that GLY was protective, (e.g., reducing clinical score and MPO). However, unexpectedly, we found that neutral pH GLY optimally reduced plate count over acidic treatment. Our data parallels other studies in a rabbit model of *S. aureus* infection, where protection was shown after topical GLY-based nanodrug formulations containing thymol (pH 7.4) (Song et al., 2020), or hesperetin (pH 7.0) (Zhang et al., 2021). Similarly, in a transgenic hepatitis B mouse model, treatment with GLY (pH not specified) significantly decreased the intrahepatic recruitment of neutrophils and other inflammatory cells after cytotoxic T lymphocyte injection (Sitia et al., 2007).

GLY primarily exerts its effects by inhibiting the chemoattractant and mitogenic activities of HMGB1 (Mollica et al., 2007). In this regard, GLY treatment at either acidic or neutral pH significantly reduced HMGB1and several proinflammatory molecules at the mRNA and protein levels (TNF-α and CXCL2). These data concur with previous studies from our lab where acidic pH GLY treatment effectively reduced CXCL2 expression (mRNA and protein) in KEI 1025-infected corneas (Ekanayaka et al., 2016). Similarly, GLY (pH not specified) protected rats against sepsis by inhibiting HMGB1 signaling (Zhao et al., 2017).

MDR9-infected mice were treated with a second generation fluoroquinolone, moxifloxacin (to which MDR9 is resistant, data not shown), which is at neutral pH (6.8) and more often used clinically to treat *P. aeruginosa* keratitis (Miller, 2008) than ciprofloxacin (pH 4.5). Treatment began 18 h after infection, and when GLY was combined with the moxifloxacin, its pH did not affect reduction of viable plate count or MPO. These data are similar to what has been shown for ciprofloxacin and acidic pH GLY (Hazlett et al., 2019). GLY (pH not specified) also has been shown to be effective in potentiating antibiotics such as ampicillin, rifampicin, tetracycline, and nalidixic acids against Gram-positive and Gram-negative bacteria (Dudhatra et al., 2012). Collectively, these data suggest that the pH of GLY is not critical to antibiotic effectiveness when they are used in combination.

In conclusion, at the normal, unwounded ocular surface, GLY at either pH decreased bulbar conjunctival goblet cells but did not induce changes in the normal cornea. However, in the wounded cornea, GLY at either pH delayed wound closure at an early, but not later time point, and after infection; using a non-antibiotic-resistant bacterial isolate (KEI 1025), neutral *vs*. acidic pH GLY was significantly better at bacterial killing. pH did not affect its ability to potentiate the antibiotic moxifloxacin for MDR9.

## Data Availability Statement

The raw data supporting the conclusions of this article will be made available by the authors, without undue reservation.

## Ethics Statement

The animal study was reviewed and approved by Institutional Animal Care and Use Committee of Wayne State University (IACUC-21-04-3499).

## Author Contributions

LH, SM, and MS contributed to conception and design of the study. SM, MS, DB, and AP performed the experiments. SM, MS, and DB carried out data analysis. MS and LH wrote the manuscript. All authors contributed to the article and approved the submitted version.

## Funding

This study was supported by the funding from National Institutes of Health/National Eye Institute grants R01EY016058 and P30EY04068 and Research to Prevent Blindness (unrestricted grant to Kresge Eye Institute).

## Conflict of Interest

The authors declare that the research was conducted in the absence of any commercial or financial relationships that could be construed as a potential conflict of interest.

## Publisher’s Note

All claims expressed in this article are solely those of the authors and do not necessarily represent those of their affiliated organizations, or those of the publisher, the editors and the reviewers. Any product that may be evaluated in this article, or claim that may be made by its manufacturer, is not guaranteed or endorsed by the publisher.

## References

[B1] Abdel Bar F. M. Elimam D. M. Mira A. S. El-Senduny F. F. Badria F. A. (2019). Derivatization, Molecular Docking and *In Vitro* Acetylcholinesterase Inhibitory Activity of Glycyrrhizin as a Selective Anti-Alzheimer Agent. Nat. Prod. Res. 33 (18), 2591–2599. doi: 10.1080/14786419.2018.1462177 29656653

[B2] Arase Y. Ikeda K. Murashima N. Chayama K. Tsubota A. Koida I.. (1997). The Long Term Efficacy of Glycyrrhizin in Chronic Hepatitis C Patients. Cancer 79 (8), 1494–1500. doi: 10.1002/(sici)1097-0142(19970415)79:8<1494::aid-cncr8>3.0.co;2-b 9118029

[B3] Asl M. N. Hosseinzadeh H. (2008). Review of Pharmacological Effects of Glycyrrhiza Sp. and Its Bioactive Compounds. Phytother. Res. 22 (6), 709–724. doi: 10.1002/ptr.2362 18446848 PMC7167813

[B4] Burillon C. Chiambaretta F. Pisella P. J. (2018). Efficacy and Safety of Glycyrrhizin 2.5% Eye Drops in the Treatment of Moderate Dry Eye Disease: Results From a Prospective, Open-Label Pilot Study. Clin. Ophthalmol. 12, 2629–2636. doi: 10.2147/OPTH.S186074 30587909 PMC6300369

[B5] Chakotiya A. S. Tanwar A. Narula A. Sharma R. K. (2016). Alternative to Antibiotics Against Pseudomonas Aeruginosa: Effects of Glycyrrhiza Glabra on Membrane Permeability and Inhibition of Efflux Activity and Biofilm Formation in Pseudomonas Aeruginosa and its *In Vitro* Time-Kill Activity. Microb. Pathog. 98, 98–105. doi: 10.1016/j.micpath.2016.07.001 27392698

[B6] Ciganović P. Jakimiuk K. Tomczyk M. Zovko Končić M. (2019). Glycerolic Licorice Extracts as Active Cosmeceutical Ingredients: Extraction Optimization, Chemical Characterization, and Biological Activity. Antioxidants 8 (10), 445. doi: 10.3390/antiox8100445 PMC6826613 31581512

[B7] Dudhatra G. B. Mody S. K. Awale M. M. Patel H. B. Modi C. M. Kumar A.. (2012). A Comprehensive Review on Pharmacotherapeutics of Herbal Bioenhancers. TheScientificWorldJournal 2012:637953. doi: 10.1100/2012/637953 PMC3458266 23028251

[B8] Ekanayaka S. A. McClellan S. A. Barrett R. P. Hazlett L. D. (2018). Topical Glycyrrhizin is Therapeutic for Pseudomonas Aeruginosa Keratitis. J. Ocul. Pharmacol. Ther. 34 (3), 239–249. doi: 10.1089/jop.2017.0094 29236588 PMC5899296

[B9] Ekanayaka S. A. McClellan S. A. Barrett R. P. Kharotia S. Hazlett L. D. (2016). Glycyrrhizin Reduces HMGB1 and Bacterial Load in Pseudomonas Aeruginosa Keratitis. Invest. Ophthalmol. Vis. Sci. 57 (13), 5799–5809. doi: 10.1167/iovs.16-20103 27792814 PMC5089214

[B10] Gong G. Xiang L. Yuan L. Hu L. Wu W. Cai L.. (2014). Protective Effect of Glycyrrhizin, a Direct HMGB1 Inhibitor, on Focal Cerebral Ischemia/Reperfusion-Induced Inflammation, Oxidative Stress, and Apoptosis in Rats. PloS One 9 (3), e89450. doi: 10.1371/journal.pone.0089450 24594628 PMC3942385

[B11] Hanafi M. Talebpour Amiri F. Shahani S. Enayatifard R. Ghasemi M. Karimpour A. A. (2018). Licorice Cream Promotes Full-Thickness Wound Healing in Guinea Pigs. J. Res. Pharm. 22 (1), 84–94. doi: 10.12991/jrp.2018.81

[B12] Hattori T. Ikematsu S. Koito A. Matsushita S. Maeda Y. Hada M.. (1989). Preliminary Evidence for Inhibitory Effect of Glycyrrhizin on HIV Replication in Patients With AIDS. Antiviral Res. 11 (5-6), 255–261. doi: 10.1016/0166-3542(89)90035-1 2572198

[B13] Hazlett L. D. Ekanayaka S. A. McClellan S. A. Francis R. (2019). Glycyrrhizin Use for Multi-Drug Resistant Pseudomonas Aeruginosa: *In Vitro* and *In Vivo* Studies. Invest. Ophthalmol. Vis. Sci. 60 (8), 2978–2989. doi: 10.1167/iovs.19-27200 31311033 PMC6944246

[B14] He J. Bazan H. E. (2016). Neuroanatomy and Neurochemistry of Mouse Cornea. Invest. Ophthalmol. Vis. Sci. 57 (2), 664–674. doi: 10.1167/iovs.15-18019 26906155 PMC4771196

[B15] Hobden J. A. Masinick S. A. Barrett R. P. Hazlett L. D. (1997). Proinflammatory Cytokine Deficiency and Pathogenesis of Pseudomonas Aeruginosa Keratitis in Aged Mice. Infect. Immun. 65, 2754–2758. doi: 10.1128/iai.65.7.2754-2758.1997 9199446 PMC175388

[B16] Hocaoglu A. B. Karaman O. Erge D. O. Erbil G. Yilmaz O. Bagriyanik A.. (2011). Glycyrrhizin and Long-Term Histopathologic Changes in a Murine Model of Asthma. Curr. Ther. Res. Clin. Exp. 72 (6), 250–261. doi: 10.1016/j.curtheres.2011.11.002 24648593 PMC3957157

[B17] Hou Y. Lan J. Zhang F. Wu X. (2021b). Expression Profiles and Potential Corneal Epithelial Wound Healing Regulation Targets of High-Mobility Group Box 1 in Diabetic Mice. Exp. Eye. Res. 202, 108364. doi: 10.1016/j.exer.2020.108364 33227295

[B18] Hou Y. Xin M. Li Q. Wu X. (2021a). Glycyrrhizin Micelle as a Genistein Nanocarrier: Synergistically Promoting Corneal Epithelial Wound Healing Through Blockage of the HMGB1 Signaling Pathway in Diabetic Mice. Exp. Eye. Res. 204, 108454. doi: 10.1016/j.exer.2021.108454 33497689

[B19] Huang X. Barrett R. P. McClellan S. A. Hazlett L. D. (2005). Silencing Toll-Like Receptor-9 in Pseudomonas Aeruginosa Keratitis. Invest. Ophthalmol. Vis. Sci. 46 (11), 4209–4216. doi: 10.1167/iovs.05-0185 16249500

[B20] Isbrucker R. A. Burdock G. A. (2006). Risk and Safety Assessment on the Consumption of Licorice Root (Glycyrrhiza Sp.), its Extract and Powder as a Food Ingredient, With Emphasis on the Pharmacology and Toxicology of Glycyrrhizin. Regul. Toxicol. Pharmacol. 46 (3), 167–192. doi: 10.1016/j.yrtph.2006.06.002 16884839

[B21] Liu Y. Xiang J. Liu M. Wang S. Lee R. J. Ding H. (2011). Protective Effects of Glycyrrhizic Acid by Rectal Treatment on a TNBS-Induced Rat Colitis Model. J. Pharm. Pharmacol. 63 (3), 439–446. doi: 10.1111/j.2042-7158.2010.01185.x 21749393

[B22] Li Y. J. Wang L. Zhang B. Gao F. Yang C.,. M. (2019). Glycyrrhizin, an HMGB1 Inhibitor, Exhibits Neuroprotective Effects in Rats After Lithium-Pilocarpine-Induced Status Epilepticus. J. Pharm. Pharmacol. 71 (3), 390–399. doi: 10.1111/jphp.13040 30417405

[B23] Mencucci R. Favuzza E. Menchini U. (2013). Assessment of the Tolerability Profile of an Ophthalmic Solution of 5% Glycyrrhizin and Copolymer PEG/PPG on Healthy Volunteers and Evaluation of its Efficacy in the Treatment of Moderate to Severe Blepharitis. Clin. Ophthal. 7, 1403–1410. doi: 10.2147/OPTH.S47657 PMC3712082 23874081

[B24] Miller D. (2008). Review of Moxifloxacin Hydrochloride Ophthalmic Solution in the Treatment of Bacterial Eye Infections. Clin. Ophthalmol. 2, 77–91. doi: 10.2147/opth.s1666 19668391 PMC2698721

[B25] Mollica L. De Marchis F. Spitaleri A. Dallacosta C. Pennacchini D. Zamai M.. (2007). Glycyrrhizin Binds to High-Mobility Group Box 1 Protein and Inhibits its Cytokine Activities. Chem. Biol. 14 (4), 431–441. doi: 10.1016/j.chembiol.2007.03.007 17462578

[B26] Moon M. M. Hazlett L. D. Hancock R. E. Berk R. S. Barrett R. (1988). Monoclonal Antibodies Provide Protection Against Ocular Pseudomonas Aeruginosa Infection. Invest. Ophthalmol. Vis. Sci. 29 (8), 1277–1284. 2843483

[B27] Mostafa S. Hoda I. Elham M. Amal M. (2004). Some Effects of Glycyrrhiza Glabra (Licorice) Roots Extract on Male Rats. Egyptian J. Nat. Toxins 1, 83–94.

[B28] Ni Y. F. Kuai J. K. Lu Z. F. Yang G. D. Fu H. Y. Wang J.. (2011). Glycyrrhizin Treatment Is Associated With Attenuation of Lipopolysaccharide-Induced Acute Lung Injury by Inhibiting Cyclooxygenase-2 and Inducible Nitric Oxide Synthase Expression. J. Surg. Res. 165 (1), e29–e35. doi: 10.1016/j.jss.2010.10.004 21074783

[B29] Nishimoto Y. Hisatsune A. Katsuki H. Miyata T. Yokomizo K. Isohama Y. (2010). Glycyrrhizin Attenuates Mucus Production by Inhibition of MUC5AC Mrna Expression *In Vivo* and *In Vitro.* J. Pharmacol. Sci. 113 (1), 76–83. doi: 10.1254/jphs.09344fp 20453436

[B30] Ojha S. Javed H. Azimullah S. Abul Khair S. B. Haque M. E. (2016). Glycyrrhizic Acid Attenuates Neuroinflammation and Oxidative Stress in Rotenone Model of Parkinson’s Disease. Neurotox. Res. 29 (2), 275–287. doi: 10.1007/s12640-015-9579-z 26607911

[B31] Okuma Y. Liu K. Wake H. Liu R. Nishimura Y. Hui Z. (2014). Glycyrrhizin Inhibits Traumatic Brain Injury by Reducing HMGB1-RAGE Interaction. Neuropharmacology 85, 18–26. doi: 10.1016/j.neuropharm.2014.05.007 24859607

[B32] Omar H. R. Komarova I. El-Ghonemi M. Fathy A. Rashad R. Abdelmalak H. D.. (2012). Licorice Abuse: Time to Send a Warning Message. Ther. Adv. Endocrinol. Metab. 3 (4), 125–138. doi: 10.1177/2042018812454322 23185686 PMC3498851

[B33] Sinha S. K. Prasad S. K. Islam M. A. Gurav S. S. Patil R. B. Al Faris N. A.. (2020). Identification of Bioactive Compounds From Glycyrrhiza Glabra as Possible Inhibitor of SARS-Cov-2 Spike Glycoprotein and non-Structural Protein-15: A Pharmacoinformatics Study. J. Biomol. Struct. Dyn. 18, 1–15. doi: 10.1080/07391102.2020.1779132 PMC7309308 32552462

[B34] Sitia G. Iannacone M. Müller S. Bianchi M. E. Guidotti L. G. (2007). Treatment With HMGB1 Inhibitors Diminishes CTL-Induced Liver Disease in HBV Transgenic Mice. J. Leukoc. Biol. 81 (1), 100–107. doi: 10.1189/jlb.0306173 16935945

[B35] Song K. Yan M. Li M. Geng Y. Wu X. (2020). Preparation and *In Vitro*-*In Vivo* Evaluation of Novel Ocular Nanomicelle Formulation of Thymol Based on Glycyrrhizin. Colloids Surf. B. Biointerfaces. 194, 111157. doi: 10.1016/j.colsurfb.2020.111157 32505061

[B36] Sun Y. Chen H. Dai J. Wan Z. Xiong P. Xu Y.. (2018). Glycyrrhizin Protects Mice Against Experimental Autoimmune Encephalomyelitis by Inhibiting High-Mobility Group Box 1 (HMGB1) Expression and Neuronal HMGB1 Release. Front. Immunol. 9, 1518. doi: 10.3389/fimmu.2018.01518 30013568 PMC6036111

[B37] Wang W. Zhao F. Fang Y. Li X. Shen L. Cao T.. (2013). Glycyrrhizin Protects Against Porcine Endotoxemia Through Modulation of Systemic Inflammatory Response. Crit. Care 17 (2), R44. doi: 10.1186/cc12558 23497622 PMC3672474

[B38] Williams R. N. Paterson C. A. Eakins K. E. Bhattacherjee P. (1982-1983). Quantification of Ocular Inflammation: Evaluation of Polymorphonuclear Leucocyte Infiltration by Measuring Myeloperoxidase Activity. Curr. Eye Res. 2 (7), 465–470. doi: 10.3109/02713688208996350 6303695

[B39] Yang R. Yuan B. C. Ma Y. S. Zhou S. Liu Y. (2017). The Anti-Inflammatory Activity of Licorice, a Widely Used Chinese Herb. Pharm. Biol. 55 (1), 5–18. doi: 10.1080/13880209.2016.1225775 27650551 PMC7012004

[B40] Zaki A. A. El-Bakry H. Fahmy A. A. (2005). Effects of Licorice on Wound Healing in Rabbits. Egyptian J. Hosp. Med. 20, 58–65. doi: 10.21608/ejhm.2005.18094

[B41] Zhang F. Chen H. Lan J. Song K. Wu X. (2021). Preparation and *In Vitro*/*In Vivo* Evaluations of Novel Ocular Micelle Formulations of Hesperetin With Glycyrrhizin as a Nanocarrier. Exp. Eye. Res. 202, 108313. doi: 10.1016/j.exer.2020.108313 33080302

[B42] Zhao F. Fang Y. Deng S. Li X. Zhou Y. Gong Y.. (2017). Glycyrrhizin Protects Rats From Sepsis by Blocking HMGB1 Signaling. Biomed. Res. Int. 2017, 9719647. doi: 10.1155/2017/9719647 28484719 PMC5412259

[B43] Zhou Y. Wang T. Wang Y. Meng F. Ying M. Han R.. (2020). Blockade of Extracellular High-Mobility Group Box 1 Attenuates Inflammation-Mediated Damage and Haze Grade in Mice With Corneal Wounds. Int. Immunopharmacol. 83, 106468. doi: 10.1016/j.intimp.2020.106468 32279044

